# Diseases of Respiratory Organs

**Published:** 1905-10-07

**Authors:** 


### Progress in Medicine and Surgery.

### DISEASES OF RESPIRATORY ORGANS.

**The Treatment of Hæmoptysis by Nitrite of Amyl.**—Francis Hare of Brisbane [1] points out that in order to stop hæmorrhage the first step is to reduce the difference between the pressures in the vessel and outside it. The pressure outside could theoretically be raised by injecting air into the pleura, or by favouring the accumulation of extravasated blood in the lung. The reduction of the pressure in the vessel may be attained by (*a*) giving astringents or ergot to constrict the pulmonary vessels, but clinically the results are unsatisfactory, and it is doubtful whether any drug really causes contraction of those arterioles. On the other hand, to reduce pressure by (*b*) reducing the force of the heart, except by enforcing rest or giving moderate doses of aconite, is dangerous, and the only other method (*c*) of lowering intra-vascular pressure is to dilate the systemic arteries as by the use of the nitrites. These theoretical indications were substantiated by actual trial. In seventeen attacks nitrite of amyl was given to nine patients. In fifteen, bleeding ceased at once and in the two others within ten minutes. In half the patients no recurrence took place, and in most of the others there was a cessation for some hours. A single administration too shows the patient that by the rapidity of its action immediate relief can be obtained, and this knowledge has of itself a very quieting effect on the heart. On the whole the results seem remarkably good. H. C. Colman [2] reports a similar success and also tried nitrite of amyl in menorrhagia where ergot and other measures had failed. With rest in bed a single dose checked the excess at each period, giving marked relief. Whether Hare's explanation of the process is correct or no, the method is clearly not one which can be neglected.

**Tuberculin Treatment.**—Tuberculin has by no means proved to be the utter failure which it at one time appeared to be. It has still an important field for utility both in diagnosis and treatment. Cranston Low [3] gives an account of its routine employment in Neisser's skin clinic at Breslau, where in hundreds of cases no instance of generalised tuberculosis has followed its use. If there is a severe affection of the lungs Neisser does not use it, but otherwise the dose commences with $\frac{1}{100}$ or $\frac{1}{10}$ of a milligramme. This is followed at intervals of 48 hours by doses of half, one, five, and ten millegrammes until a reaction occurs. This may be general with a rise of temperature of 2° or 3° C., or may be local with signs of inflammation extending some distance beyond the diseased area. The outer part of the reaction zone in skin affections contains a number of minute tubercular nodules and though it appears perfectly normal before the reaction and afterwards, it must be included in the subsequent treatment. In tuberculous patients various forms of eruption often appear later on, but never in other persons. The local reaction is of more value in diagnosis than the general one, and Neisser asserts that a lesion is tubercular when a typical local reaction occurs and not otherwise, but no hard-and-fast line can be drawn between the lowest dose which will give a general reaction in tuberculous and non tuberculous patients. This view is not accepted by others, and many authorities consider that a general reaction with one-tenth or one half milligramme is a reliable indication of tuberculosis when given with proper precautions. Indeed, though a fixed limit cannot be given, the reaction to a small dose will often enable us to say whether a lesion is tubercular or otherwise. In the endeavour to improve the results of sanatorium methods, and as an alternative treatment for those who cannot avail themselves of them or fail to recover under them, the use of tuberculin has been gradually put on a sound basis, and it has been certainly shown to have a definite value. Batty Shaw [4] gives an account of the present state of the problem, and compares the various methods employed. Besides Koch's old tuberculin, his Tuberculin R., and his new tuberculin or bacillary emulsion, there are a number of other preparations of a similar character. Several attempts have also been made to utilise the tubercle bacilli of birds and cold-blooded animals as a protective vaccine, and Moeller even injected himself with a culture of human bacilli which had been passed through the slowworm as a method of producing an attenuated form. Others have obtained sera by the injection of human bacilli into immune animals, such as goats, dogs, and horses. To this class belong Marmorek's serum and Maragliano's. It has been objected that the human being never does acquire immunity, but Wright has finally shown that the defensive power of the body can be raised without doubt and that the rise of this power can be measured. An additional method is the use of improved antistreptococcic serum to overcome the secondary infections which give rise to so much of the fever and other symptoms in phthisis. The results of tuberculin treatment in 656 early cases collected by Pottenger averaged 91 per cent. of cures in the technical sense, while out of 611 similar cases treated without tuberculin the cures only reached 62 per cent. Again, taking a large number of cases of various degrees of severity, Pottinger found that 44·9 per cent. were reported as apparently cured under tuberculin and only 13 per cent. when it was not used. As to the value of sera on the other hand, though many satisfactory results have been reported from their employment, it is not possible as yet to give such a comprehensive estimate of their utility, but as Shaw remarks the evidence in favour of tuberculin shows, at any rate, that it materially improves the results of ordinary treatment. Goetsch lays down the following rules for the use of the old tuberculin and of the bacillary emulsion. The patient should not be suffering from much fever; the dose of tuberculin must not be increased unless the last dose gave no reaction; the patient must be kept in bed on the day of injection and on the next day; the next dose, if a moderate reaction has taken place, should be the same as the last; the dose after a severe reaction should be diminished, and the interval of time between the doses should be four to eight days; the intervals if no reactions take place, should be two clear days. The course of injections may be repeated at intervals for months or years. Kohler and Behr[5] point out that in many consumptives the temperature is so unstable that a mere suggestion is sufficient to cause a rise, so that in the administration of tuberculin we may easily be misled. They gave to certain patients an injection of distilled water and for others they used an empty syringe. The result was that 21 per cent. showed a rise of temperature, the average being one degree, but as much as four degrees was noted, and in the majority the rise took place at the hour suggested.

[1] Clinical Jour., March 22. [2] Scottish Med. and Surg. Jour., May. [3] Ibid. [4] Lancet, April 8, and Clinical Jour., May 3. [5] Med. Chronicle, April.

(*To be continued.*)

